# Optimal foot skin care for diabetes-related foot ulcer prevention: scoping review

**DOI:** 10.1007/s13340-025-00814-0

**Published:** 2025-03-27

**Authors:** Makoto Oe, Amika Yamada, Erlin Ifadah

**Affiliations:** 1 Faculty of Health Sciences, Institute of Medical, Pharmaceutical and Health Sciences, Kanazawa University, 5-11-80, Kodatsuno, Kanazawa, Ishikawa Japan; 2 Division of Health Sciences, Graduate School of Medical Sciences, Kanazawa University, 5-11-80, Kodatsuno, Kanazawa, Ishikawa Japan; 3 Nursing Department, Faculty of Health, Respati Indonesia University, Jl. Bambu Apus I No 3, Cipayung, Jakarta Timur, Indonesia

**Keywords:** Diabetic foot ulcer, Dry skin, Moisturizer, Urea, Xerosis

## Abstract

**Aim:**

In patients with diabetes, autonomic neuropathy leads to reduced sweating, which can lead to dry skin, and if the condition worsens, these can progress from foot fissures to ulcers. Although foot skin care is important in patients with diabetes to prevent diabetes-related foot ulcers, there are no detailed guidelines that provide specific methodology for achieving this goal. This scoping review aimed to clarify what is known in the literature regarding foot skin care for dry skin for patients with diabetes and propose optimal foot skin care to help prevent diabetes-related foot ulcers.

**Methods:**

Literature databases were searched and two independent researchers screened the articles according to the inclusion criteria and then extracted the data. To be included in the analysis, all reports had to be original articles/case studies, studies involving human subjects, and studies on foot skin care for dry skin for patients with diabetes.

**Results:**

Seven studies met the inclusion criteria. Findings showed that application of a moisturizer, especially a cream containing urea or a cream containing 15% glycerol, liquid, and 10% soft paraffin twice a day for at least two weeks, could help relieve dry feet.

**Conclusion:**

Establishing optimal foot skin care for patients with diabetes may require further studies that examine the frequency and long-term effects of foot skin care interventions, with the ultimate outcome focused on the development of diabetes-related foot ulcers.

## Introduction

Diabetes-related foot ulcer is defined as a foot ulcer in a patient with current or previously diagnosed diabetes mellitus, and which is usually accompanied by peripheral neuropathy and/or peripheral artery disease in the lower extremity [1]. This is one of the known serious diabetic complications, with a reported annual incidence and lifetime prevalence rate of 2–4% and 19–34%, respectively [2, 3]. It was reported that by 12 months, 65.7% of the patients had healed without amputation [4], while 5% were required to undergo a major amputation within 1 year [5]. Diabetes-related foot ulcer affects not only the patient’s quality of life, [6, 7] but also is an economic burden on society [8, 9]. In 2021, there were 537 million adults (20–79 years) who were living with diabetes [10]. This number is predicted to rise to 643 million by 2030 and to 783 million by 2045. Due to the global increase in the number of patients with diabetes, there is also an urgent need to establish preventive measures for diabetes-related foot ulcer.

To prevent diabetes-related foot ulcer, it has been recommended that at-risk feet, such as a foot having a loss of protective sensation and being identified as having peripheral artery disease be regularly observed by healthcare professionals [11]. In addition, it is recommended that patients with at-risk feet be treated for lesions that could potentially lead to foot ulcers in addition to being educated on issues regarding skin care. In patients with diabetes, autonomic neuropathy leads to reduced sweating, which can lead to dry skin, and if the condition worsens, these can progress from foot fissures to ulcers [3, 12, 13]. Foot skin care is essential for the prevention of foot lesions such as dry skin, with the basic principles of foot skin care including both cleaning and moisturizing of the feet, in addition to careful observation of the feet.

### Rationale for the review

With regard to prevention of the diabetes-related foot ulcers, current guidelines do not discuss specific foot skin care [14, 15], nor do they make any recommendations on the cleaning and moisturizing through the use of specific methods on how to care for the feet, such as frequency, or the type, and amount of cleansers and moisturizers that should be used [11, 16]. Although many different types of cleansers and moisturizers have been developed and marketed in recent years, there has yet to be a unified view on which cleansers and moisturizers are appropriate for use as foot skin care for patients with diabetes.

### Review objectives

The objectives of this scoping review were as follows: (a) to clarify what is known in the literature regarding foot skin care for dry skin for patients with diabetes, (b) to identify the research gaps and directions for future research, and (c) to propose optimal foot skin care to prevent diabetes-related foot ulcers.

### Review questions

Review questions were as follows: (a) what is effective foot skin care that should be used for patients with diabetes? and (b) what type of research is required to determine the optimal foot skin care that can be used to prevent diabetes-related foot ulcers?

## Methods

### Protocol and registration

A scoping review was performed according to the Preferred Reporting Items for Systematic reviews and Meta-Analyses extension for scoping reviews ([PRISMA]-ScR) [17]. The review protocol was not registered.

### Eligibility criteria

This review focused on the utilization of cleaning and moisturizing care as foot skin care for dry skin. We included studies involving participants of all ages, regardless of the clinical setting, and publication year. Studies that did not involve human subjects, such as animals or cells, were excluded. However, studies were included in this review even when the subject pool was not limited to patients with diabetes, provided that there was a statement that patients with diabetes were included in the analyses. The literature search was limited to articles that were published in English. Originally published articles and case reports were included in this scoping review. Proceedings, conference abstracts, letters to the editor, editorials, guidelines, protocols, literature reviews, and meta-analyses were excluded.

### Information sources

To identify potentially relevant documents, the PubMed and CINAHL databases were searched on September 23, 2024. The search strategies were created through team discussion. The final search results exported to Rayyan (Qatar Computing Research Institute, Doha, Qatar), with all duplicates eliminated by one of the researchers (M.O.) [18].

### Search

The search formula was as follows: ((diabetes mellitus) OR (diabetic mellitus)) AND (skin care) Filtered by Humans, English.

### Selection of sources of evidence

Two independent researchers (M.O. and A.Y.) read the titles and abstracts and excluded articles that did not meet the eligibility criteria. Subsequently, the full-text articles that remained after the initial evaluation were then screened according to the inclusion criteria by the same two independent researchers (M.O. and A.Y.). Disagreements regarding the study selection were resolved through discussion.

### Data charting process

A data charting form was developed by one author (M.O.) to determine the data items to be extracted. Data were extracted by a single author (M.O.) and verified by the co-authors (A.Y. and E.I.). Discrepancies in the extracted data were resolved through a discussion among the three authors (M.O., A.Y., and E.I.).

### Data items

The following information was extracted: (a) study authors, year of publication, and country; (b) study design/participants; (c) participant characteristics; (d) foot skin care; (e) main outcome; (f) key findings for review questions.

### Summary of results

A single author (M.O.) summarized the content, while the co-author (E.I.) double-checked the content. The three reviewers (M.O., A.Y., and E.I.) discussed the results when there was a lack of consensus.

## Results

### Selection of analysis sources

**Fig. 1:**
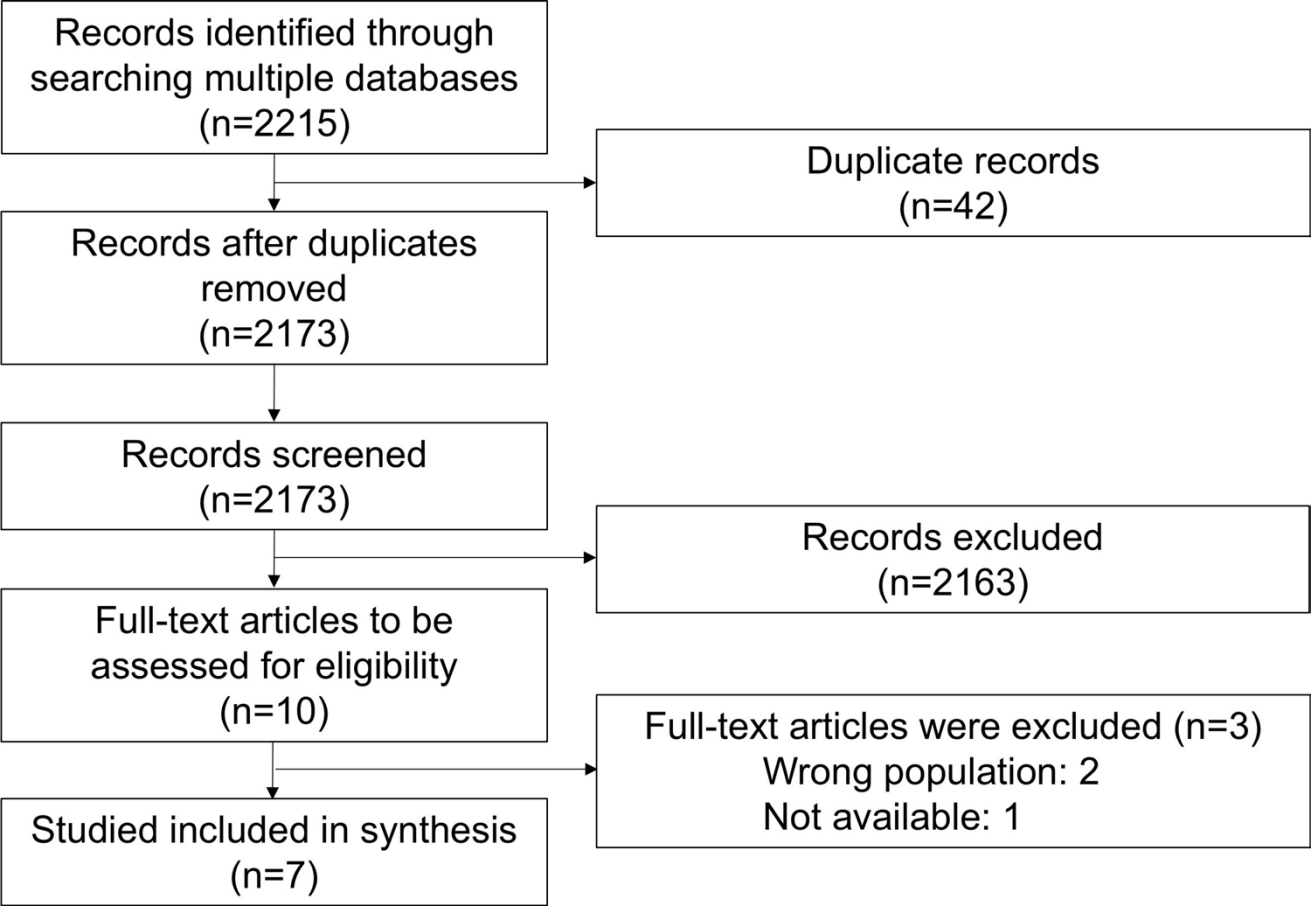
Flowchart of this scoping review

The initial search yielded 2215 studies. As there were 42 duplicate records, 2173 studies were eligible for the first screening. After screening the titles and abstracts, 2163 papers were excluded. Among the ten remaining papers, three were ineligible after full-text screening: two studies included the wrong population and one study was not available. As a result, seven studies met the inclusion criteria and were included in the scoping review [19–25]. Figure 1 shows the PRISMA flowchart used for this review.

### Characteristics of the evidence sources

Tables 1 summarizes the characteristics of the included studies. Six were randomized-controlled trials [19, 20, 22–25] and one was a non-randomized-controlled trial [21]. The included studies were published between 2011 and 2024 and were conducted by research teams in Spain, Germany, USA, France, and Italy. Six studies included only patients with diabetes, while one study included subjects both with and without diabetes.

**Table 1:** Summary of the data collected from studies on foot skin care for patients with diabetes

Author/year/country	Study design/participants	Characteristics	Foot skin care	Main outcome	Key findings for review questions
Ramírez et al., 2024, Spain [19]	Randomized-controlled trial, *n* = 21	Patients with diabetic foot syndrome	To apply the assigned 10% urea cream to the dorsum and sole of the designated foot at the rate of 2 mL per day for 30 days Pharmacy cream group vs. supermarket cream group	Questionnaire for the evaluation of injury risk and skin quality of the foot of the patient with diabetic foot syndrome	The skin quality was significantly improved between before and after application (4.22 ± 2.72, 3.27 ± 2.14 points, respectively, *p* < 0.001). Differences in the improvement were not significant between the two groups (*p* = 0.395)
Fitzner et al., 2023, Germany [20]	Randomized-controlled trial, *n* = 60	Subjects aged 35 and 75 years old; had healthy skin, half of whom had a diagnosis of diabetes	To apply the test products (creams) to the test area (dorsal and planter) twice daily for 4 weeks Untreated vs. neutral test product (pH 6.9–6.25) vs. acidic test product (pH 4.5–4.63) vs. alkaline test product (Batch 1: pH 8.5–6.6, Batch 2: pH 8.5–8.14)	Skin pH and hydration	Only slight increases in the skin pH were observed in treatment groups. In the case of the neutral test product and acidic test product, no significant differences were observed. The mean pHs for all treatment groups remained well within the physiological pH range of the skin After 29 days of measurement, all three formulations increased the hydration of the skin as compared with the average baseline measurement. There was no significant difference in skin hydration values between days 15 and 29
Kirsner et al., 2023, USA [21]	Non-randomized-controlled trial, *n* = 528	Subjects with diabetes-related xerosis The xerosis sites studied included the legs (76%) and feet (58%)	To apply the ceramide-containing cleanser and moisturizing cream twice a day to the areas of xerosis for 28 days	Dry skin classification scale	All parameters (roughness/scaling, erythema, and fissures) of the dry skin classification scale significantly improved from baseline to the end of the study (*p* < 0.001)
Glonek et al., 2022, USA [22]	Randomized-controlled trial, *n* = 54	Patients with diabetes and dry feet	To apply the assigned skin cream to the right or left foot twice daily for 6 weeks Skin cream containing plant-based 0.05% anionic polar phospholipid technology vs. a mineral oil hydrocarbon-based skin cream	Diabetic foot care grading system	In all parameters (dryness, fissures, erythema, itching, and composite score), a significant effect for the observation times was observed at the 0.05 level of significance. However, there was no difference due to the effect of the treatment group or the interaction of the treatment group with time
Martini et al., 2017, France [23]	Randomized-controlled trial, *n* = 57	Patients with diabetes	To apply the assigned cream twice daily for 28 days A cream containing 15% glycerol, liquid and 10% soft paraffin, glycerol monostearate, stearic acid, polydimethylcyclosiloxane, silicone oil, macrogol 600, trolamine, propyl parahydroxybenzoate, and purified water vs. its vehicle	Xerosis assessment scale and overall skin score	The xerosis assessment scale score decreased to 3.2 ± 2.6 points with the cream and 4.1 ± 2.3 with the vehicle (*p* = 0.001). Improvement was observed from day 14 (*p* = 0.012). Compared with the vehicle, the cream also significantly improved the overall skin score
Federici et al., 2012, Italy [24]	Randomized-controlled trial, *n* = 40	Patients with type 2 diabetes	To apply the assigned cream twice daily for 28 days, which contained 5% urea, arginine and carnosine-based cream vs. glycerol-based emollient cream (control)	Dryness area severity index score	After 4 weeks, as compared with the control group, there was a significantly lower dryness area severity index score in the 5% urea, arginine, and carnosine-based cream treated group (0.2 vs. 1.0; *p* = 0.048)
Garrigue et al., 2011, France [25]	Randomized-controlled trial, *n* = 54	Patients with type 1 or 2 diabetes and moderate-to-severe xerosis	To apply the assigned skin cream to the right or left foot twice daily for four weeks The cream contained 10% glycerine, 5% urea, 1% lactic acid, and 8% paraffin vs. the placebo	Xerosis assessment scale	There was a dramatic decrease in xerosis assessment scale score that was more marked with the cream containing 10% glycerine, 5% urea, 1% lactic acid, and 8% paraffin as compared to the placebo observed from day 14 (38.1% vs. 20.9%, *p* < 0.0001), reaching 61.9% vs. 34.9% at day 28 (*p* < 0.0001)

### Analysis of the findings

Three of the studies examined the effects of using creams with urea. Ramírez et al. demonstrated that the application of 10% urea cream at 2 mL per day for 30 days significantly improved skin quality [19]. Federici et al. reported that the dryness area severity index score was significantly lower when applying 5% urea, arginine, and carnosine-based cream twice a day for 28 days as compared to the application of glycerol-based emollient cream [24]. Garrigue et al. reported that application of a cream containing 10% glycerine, 5% urea, 1% lactic acid, and 8% paraffin twice a day for 4 weeks reduced the xerosis assessment scale scores after 14 days as compared to placebo [25].

One of the studies examined the effects of cleanser and creams with ceramide. Kirsner et al. tested the effectiveness of using the ceramide-containing cleanser and moisturizer twice a day for 28 days [21]. There was a significant improvement of all parameters (roughness/scaling, erythema, and fissures) of the dry skin classification scale from baseline to the end of the study.

Martini et al. tested the effects of using a cream that contained 15% glycerol, liquid and 10% soft paraffin, glycerol monostearate, stearic acid, polydimethylcyclosiloxane, silicone oil, macrogol 600, trolamine, propyl parahydroxybenzoate, and purified water after application of the cream twice a day for 28 days [17]. After 14 days, there was a significant improvement in the xerosis assessment scale score in both groups. Compared with the vehicle, there was also a significant improvement in the overall skin score for the cream.

Glonek et al. evaluated the effectiveness of skin cream containing plant-based 0.05% anionic polar phospholipid technology that was applied twice daily for 6 weeks to the left and right feet of the subjects. There was a significant effect observed for all parameters (dryness, fissures, erythema, itching, and composite score) at each of the evaluations times. However, there was no difference over time due to the effect or the interaction of the treatment groups [22].

Fitzner et al. evaluated three creams that had a different pH twice a day for 4 weeks (untreated vs. neutral test product vs. acidic test product vs. alkaline test product) [20]. The mean pHs for all treatment groups remained well within the physiological pH range of the skin. After 29 days of measurement, all three formulations increased the hydration of the skin as compared to the average baseline measurement. There was no significant difference in the skin hydration values between days 15 and 29.

## Discussion

### Summary of findings

In this scoping review, we identified six randomized-controlled trials and a non-randomized-controlled trial on foot skin care for patients with diabetes, all of which were published between 2011 and 2024. Based on our findings, we recommend applying a moisturizer, especially a cream containing urea, or a cream containing 15% glycerol, liquid and 10% soft paraffin twice a day for at least two weeks for dry skin. However, there were no studies on skin care that utilized the occurrence of diabetes-related foot ulcer as an outcome.

### Studies on dry skin

In general, moisturizers restore skin hydration through mechanisms of occlusion, humectancy, hydrophilic matrices, and photoprotection [26]. Moisturizer formulations include creams, lotions, ointments, and serums, with creams and ointments, which are especially used for the feet. Ointments are anhydrous semisolid preparations composed of fats, waxes, animal and plant oils, and hydrocarbons, and they are also waterproof. However, these possess poor esthetics, because they are sticky and have a tendency to stain clothing. In contrast, creams are emulsions containing hydrophilic and hydrophobic ingredients, with oil-in-water emulsions the most popular for moisturizer use. Cream-type moisturizers were evaluated in all of the studies reviewed in the present study.

In addition, moisturizing ingredients include petrolatum, silicone, ceramides, fatty acids, lipid trilayers, and natural moisturizing factors such as urea, and lactic acid [26]. In most studies, dry skin improved not only in conjunction with the moisturizer tested, but also in the controls. These results may indicate that applying some kind of moisturizer may be effective in treating dry feet in patients with diabetes. This review also found that only creams containing 5% urea, arginine and carnosine [24], cream containing 10% glycerine, 5% urea, 1% lactic acid, and 8% paraffin [25], and cream containing 15% glycerol, liquid, and 10% soft paraffin, [23] showed an effect as compared to that observed in the controls. By forming a lipid layer on the skin surface, glycerine and paraffin help to improve skin barrier function and prevent dehydration, thereby reducing transepidermal water loss [25]. In contrast, glycerine, urea, and lactic acid are part of a natural moisturizing factor, which is a group of water-soluble substances that are responsible for maintaining water within the keratinized epidermal layers of the skin. Urea functions as a natural endogenous humectant by replacing water in low humidity conditions and maintaining a fluidic stratum corneum [27]. The properties of urea are concentration-dependent, with moisturizing effects observed at 5%, with a desquamation action at 20% and keratolytic action at 40% [25]. Lactic acid is a hygroscopic water-soluble compound characterized by its high water-capturing ability and excellent substantivity on cutaneous proteins, thereby enabling a long-lasting efficacy. In the experiment that utilized urea, arginine and carnosine-based cream, the effect of urea and carnosine on advanced glycosylation end products, the increase in skin hydration due to increased aquaporin synthesis by urea, and the improvement of the microcirculation by arginine were all considered to be the factors that improved dry skin [24]. Thus, if greater effectiveness is required, the use of moisturizers containing these ingredients would be recommended.

While most of the verified application conditions were performed twice a day, and the longest usage period was 42 days, there was no verification conducted that compared the frequency of the application. Thus, more effective care methods could potentially be discovered by further verifying the frequency of the application. Moreover, although it is recommended that these applications be performed for more than 2 weeks based on the previous reports that the beneficial effect appears after about 14 days, this recommendation appears to be based on naturally occurring changes, as the average turnover of fully keratinized cells in normal human epidermis is 14 days [28]. Therefore, the long-term effects and adverse events that occur after 42 days remain unknown and will need to be verified in future studies. On the other hand, considering the mechanism of epidermal turnover, the continuity of foot skin care will undoubtedly affect outcomes. Factors related to the continuity of foot skin care, such as usability and cost, will also need to be investigated in the future.

While the present review evaluated the previous studies that examined several moisturizers, we did not find any previous studies that examined cleansers alone. Although cleansers that aim to have moisturizing effects have also been developed, future studies will need to be conducted to definitively evaluate their effectiveness.

### Limitations

To obtain a broad range of knowledge on this subject, this review included papers that analyzed data on subjects without diabetes [20] and evaluations on subjects that involved areas other than just the foot [21]. Regarding the effects of cleansers and moisturizers containing ceramides, and creams with different pH levels, it cannot be denied that different results could potentially be obtained when the research is limited to only the feet of patients with diabetes. The cleansers and moisturizers covered in this review contain multiple active ingredients, and the results of this review do not definitively prove the effectiveness of any particular ingredient or base materials. The focus of this review was only on cleansing and moisturizing. In addition to these, the prevention of diabetes-related foot ulcers also requires self-care such as foot inspections, nail cutting, not using chemical agents or plasters to remove callus and the avoidance of walking barefoot or in only socks or in thin-soled slippers, in addition to the avoidance of wearing tight socks [16]. However, this review did not specifically evaluate these areas.

This review included only seven studies, and none of them specifically examined long-term effects, so it was not possible to suggest any effective long-term foot skin care methods. More research is needed, especially those that involve long-term foot skin care interventions.

## Conclusions

In this scoping review, we examined the studies on foot skin care for dry skin in patients with diabetes. We found that applying a moisturizer, especially a cream containing urea or a cream containing 15% glycerol, liquid, and 10% soft paraffin twice a day for at least 2 weeks can help relieve dry feet. Establishing optimal foot skin care for patients with diabetes may require further studies that examine both the frequency and long-term effects of these foot skin care interventions, with a focus on the development of a diabetes-related foot ulcer as the ultimate outcome.
